# Updated Values for Molecular Diagnosis for Highly Pathogenic Avian Influenza Virus

**DOI:** 10.3390/v4081235

**Published:** 2012-08-07

**Authors:** Akira Sakurai, Futoshi Shibasaki

**Affiliations:** Department of Molecular Medical Research, Tokyo Metropolitan Institute of Medical Science, 2-1-6, Kamikitazawa, Setagaya-ku, Tokyo 156-8506, Japan; Email: sakurai-ak@igakuken.or.jp (A.S.); shibasaki-ft@igakuken.or.jp (F.S.)

**Keywords:** influenza virus, highly pathogenic avian influenza virus H5N1, molecular diagnosis

## Abstract

Highly pathogenic avian influenza (HPAI) viruses of the H5N1 strain pose a pandemic threat. H5N1 strain virus is extremely lethal and contagious for poultry. Even though mortality is 59% in infected humans, these viruses do not spread efficiently between humans. In 1997, an outbreak of H5N1 strain with human cases occurred in Hong Kong. This event highlighted the need for rapid identification and subtyping of influenza A viruses (IAV), not only to facilitate surveillance of the pandemic potential of avian IAV, but also to improve the control and treatment of infected patients. Molecular diagnosis has played a key role in the detection and typing of IAV in recent years, spurred by rapid advances in technologies for detection and characterization of viral RNAs and proteins. Such technologies, which include immunochromatography, quantitative real-time PCR, super high-speed real-time PCR, and isothermal DNA amplification, are expected to contribute to faster and easier diagnosis and typing of IAV.

## 1. Influenza Virus

Influenza is a highly contagious respiratory disease of humans, caused by negative-strand, segmented RNA viruses belonging to the family *Orthomyxoviridae* [1]. There are three different genera of influenza virus; type A, type B, and type C. Seasonal outbreaks, caused by influenza A and B viruses, constitute a global health issue, leading to morbidity, mortality, and economic losses. Influenza A viruses (IAV) are the cause of pandemics, including “Spanish influenza” (subtype H1N1 in 1918), “Asian influenza” (subtype H2N2 in 1957), “Hong Kong influenza” (subtype H3N2 in 1968), and A/H1N1pdm (subtype H1N1 in 2009). The worst of these pandemics was “Spanish influenza”, which was reported to cause 30,000,000–60,000,000 deaths worldwide and 390,000 deaths in Japan [2,3]. IAV is classified into 16 HA and 9 NA subtypes, based on the antigenicity of two viral surface proteins—hemagglutinin (HA) and neuraminidase (NA). Almost all possible combinations of HA and NA have been isolated from aquatic birds, poultry, and other avian species. In humans and other mammals, limited subtypes of IAVs have been detected [4]. IAV contains eight gene segments encoding the corresponding viral protein(s). Pandemic viruses are generated by the rearrangement (reassortment) of viral RNA segments in cells infected with two different strains of IAV [5]. Thus, reassortment can theoretically result in 256 (2^8^) different genotypes, and is a key source of pandemic viruses. Avian and human IAVs can generally infect swine, and generate pandemic IAVs by reassortment [5]. 

Avian IAVs are differentiated on the pathogenicity in chickens between Highly pathogenic avian influenza (HPAI) and low pathogenic avian influenza (LPAI) viruses [6,7]. In contrast to mild symptoms caused by LPAI viruses, HPAI viruses cause significant mortality in chickens. All HPAI virus reported belong to H5 or H7 subtypes, but only small amount of these subtypes are HPAI viruses. The HA_0_ cleavage site plays a critical role in the pathogenicity of avian IAVs [8,9]. Cleavage of HA precursor HA_0_ is required to fusion between host and viral membrane [10]. Thus, distribution of host protease that can cleave HA_0_ is critical for the tropism [9]. HA_0_ of LPAI virus is cleaved by extracellular serine protease, meaning the spreading ability is limited to existence of available protease. On the other hand, that of HPAI virus are ubiquitously cleaved because the cleavage site contains multi-basic sequence (MBS), which is targeted by intracellular endoproteases, including PC6 and furin [8,9]. Thus, HPAI virus gains the ability to infect various cell types and cause systemic infections. 

HPAI H5N1 viruses were the cause of the first lethal human infection in Hong Kong in 1997 [11,12,13]. This constituted the first definitive transmission of avian IAVs to humans, indicating that the HPAI H5N1 virus is able to infect humans directly, without prior reassortment in swine. The HPAI H5N1 virus in Hong Kong contained an *HA* gene that was derived from A/goose/Guandong/1/96 (H5N1; GS/GD/1/96-like virus); the remaining seven segments were derived from A/teal/Hong Kong/W312/97 (H6N1; W312-like virus) [14,15]. The *HA* genes of HPAI H5N1 viruses belongs to GS/GD/1/96-linage and all HAs of HPAI H5N1 virus has a MBS (RRKKR) at the cleavage site [16]. During 2001 and 2002, the HPAI H5N1 virus caused avian outbreaks with frequent reassortment [17,18]. In 2002, genotype Z of eight new H5N1 genotypes generated by reassortment became dominant [19]. Since 2003, the HPAI H5N1 virus has spread via wild birds and poultry, throughout Asia to Europe and Africa, and has infected humans exposed to infected poultry [20,21,22]. The World Health Organization (WHO) reported that the HPAI H5N1 virus has infected 592 individuals, causing 349 deaths (~59% mortality) [23].

LPAI and HPAI H7 viruses cause several outbreaks among poultry, resulting in transmission of these viruses to humans [24]. Infection of LPAI and HPAI H7 viruses to humans usually result in conjunctivitis, which have rarely been reported with other subtypes including H5N1 HPAI viruses [25,26,27,28,29]. One of the largest outbreak of HPAI H7N7 virus occurred in the Netherland in 2003. About 90 confirmed human cases of HPAI H7N7 virus infection were reported. Most of these patients presented conjunctivitis, and some of them presented mild influenza-like illness. Only one patient, a 57-year-old veterinarian, died as a result of pneumonia in combination with acute respiratory distress syndrome [26,27,29].The cleavage site of HPAI H7N7 virus contained MBS (KRRRR) [30]. 

A recent pandemic of A/H1N1pdm occurred following reassortment between two swine IAVs—triple-reassortant swine influenza virus and Eurasian-linage swine influenza virus [31]—indicating the possibility of the occurrence of an HPAI virus pandemic by similar reassortment. If this mutated HPAI virus were to acquire the ability for efficient human-to-human transmission with high pathogenicity of HPAI virus, it might pose a serious threat to human health and the global economy. To avoid this threat, there is a need for rapid diagnosis of IAVs (including avian IAVs), not only to allow early drug administration for improved medical outcomes and patient care, but also to facilitate effective containment of infected patients. In addition, surveillance of HPAI viruses in poultry and wild birds is required for outbreak management and preparation for pandemic. 

WHO and Office International des Epizooties (World Organisation for Animal Health; OIE) recommend several techniques, including enzyme-linked immunosorbent assay (ELISA), haemagglutination inhibition (HI) and neuraminidase inhibition (NI) tests, egg and tissue culture inoculation, agar gel immunodiffusion (AGID) and both real-time and conventional reverse transcriptase PCR (RT-PCR) as newer molecular methods [32,33,34,35]. As a gold standard, IAVs have been categorized using the ELISA with HA and NA subtypes [36]. However, ELISA, HI and NI depend on specific antibodies and may not be suitable for identifying newly emerging strains. Viral replication in chicken eggs and/or tissue culture can generate a large amount of virus for diagnosis or subtyping. [33,37]. However, it requires 3~7 days to grow and the condition optimized for each viral strain. AGID requires a large amount of antigen, meaning that the virus should be propagated in chicken eggs [32]. In contrast to the limitation of these classical methods, new technologies for the detection and characterization of viral RNAs and proteins have advanced dramatically in recent years. The new techniques offer surveillance and diagnosis advantages, such as rapidity, reliability, and cost-effectiveness.

## 2. Immunochromatography

**Figure 1:**
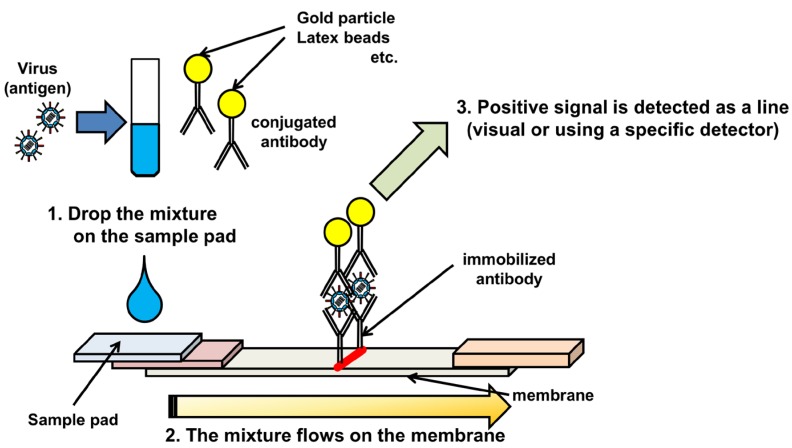
Schematic diagram of immunochromatography.

Immunochromatography (IC), an antigen-based assay completed within ~30 minutes, is an important rapid test for clinical diagnosis and surveillance of IAVs [38,39]. The principle is similar to that of sandwich ELISA. The nasal swab collected from patients is directly mixed with viral antigen-specific antibody, conjugated with colloidal gold, colored latex, or a type of enzyme (Figure 1). The mixture is dropped onto a sample pad on a nitrocellulose membrane, containing immobilized viral antigen-specific antibody for capturing the antigen. The antigen–antibody complex in the mixture flows on the membrane, and contacts with the immobilized antibody, resulting in the appearance of a line or dot as a positive signal.

IC is rapid and easy to use, but has a relatively low sensitivity [40,41]. The specificity is >90%, whereas the sensitivity is only ~60%. Thus, the technique is not suitable for the diagnosis of IAVs in the early stages of infection [41]. For detection of the H5 strain, the sensitivity of IC using H5-specific antibody was reported to be 10^4.5^–10^6^ 50 % egg infectious dose (EID_50_)/mL [42]. Recently, the sensitivity of IC has been improved by several new techniques. The simplest of these is to screen more specific and reactive antibodies. Miyagawa *et al.* [43] reported that the sensitivity of IC for the HPAI H5N1 virus using H5-specific antibodies was 10^3^ TCID_50_/mL. A second means of enhancing the sensitivity is to improve the detection system. For example, silver amplification is based on photographic technology, using silver halide crystals as a catalyst [44]. First, antigen and monoclonal antibody conjugated with gold nano particles are applied in the IC tip. Next, the buffer containing educing reagent and silver ions flows on the membrane. The silver ions cluster around the gold particle, such that the size of the colloidal gold particle is amplified ~100-fold as a silver cluster. The IC tip is then scanned by the specific IC reader and analyzed. The sensitivity of the method to detect HA protein of the HPAI H5N1 virus was reported to be 10 times higher than that of conventional IC methods to detect NP protein of the HPAI H5N1 virus [44]. In other instance, improving the sensitivity of IC is to conjugate the antibody with fluorescent beads, Quantum dot, or other fluorescent components [45,46]. The IC strip is scanned by a fluorescent reader and analyzed. Preliminary experiments indicate that the sensitivity of IC using fluorescent bead-conjugated antibody is 50 times higher than that of conventional IC methods using colloidal gold-conjugated antibody (unpublished data). Moreover, the fluorescent IC can be quantitatively analyzed. Silver amplification and fluorescent ICs still take 30 minutes to detect IAVs and offer advantages over conventional IC methods, but require specific scanners, including an image analyzer and fluorescence reader. Nevertheless, improved IC methods are expected to play an important role in the diagnosis and surveillance of IAVs, by offering rapidity and high specificity.

## 3. Sample preparation for RNA detection systems.

RNA detection systems, including PCR-based methods, DNA amplification under isothermal conditions and DNA microarray, are required for sample preparation. Samples are collected from nasal swabs or throat lavage fluid of patients. Avian samples are collected from cloacal swabs or faeces. However, it is not limited to these samples. RNA extraction step is essential for molecular diagnoses detect RNA of IAV. The target RNA is extracted from these samples by using commercial RNA extraction kit, like QIAamp® viral RNA extraction kit (Qiagen, Hilden, Germany) using silica gel membrane technology. Crude sample such as faeces is not suitable for RNA extraction, because crude samples contain a mixture of component which could inhibit reaction of RNA detection. Dhumpa *et. al.* [47] developed a new purification system by using magnetic beads coated with monoclonal antibody against viral nucleoprotein, which interacts with viral RNA. This method can purified RNA for using RNA detection systems without RNA extraction step. RNA extraction is usually a significant time-consuming step. Some protocols are available for automated system, like QIACube® (Qiagen, Hilden, Germany), which is suitable for a large amount of samples. Magnetic silica beads-based system is more rapid and convenient than spin column method with silica gel membrane technology, because high-speed centrifuge is dispensable [48,49,50]. Yang *et. al.* [51] reported a comparison of commercial systems for RNA extraction from DNA/RNA respiratory pathogens, indcluding IAV. KingFisher mL (ThermoFisher Scientific Inc., Worcester, MA, USA) and easyMAG (bioMérieux, Marcy l’Etoile, France) protocols using magnetic beads-based technology extracted 3- to 4-fold more RNA from the human IAV than other protocols. TruTip (Akonni Biosystems, Inc., Frederick, MD, USA) is a new RNA preparation system, based on a monolithic, porous nucleic acid binding matrix embedded within a pipette tip [52]. The technique is reported to be one of the most rapid RNA extraction methods (several minutes for 8 samples), and the RNA extraction efficiency and quality are comparable to QIAamp® and easyMAG extraction system [52]. The development of RNA extraction systems provides prospects for saving time in the PCR-based diagnoses.

## 4. PCR-Based Methods

### 4.1. Reverse Transcriptase-PCR/Degenerate Reverse Transcriptase-PCR

RT-PCR is a classical technique for the rapid and accurate diagnosis and typing of RNA viruses, including IAVs [53]. For diagnosis of IAV, purified influenza A viral RNA is reverse transcribed into cDNA by a reverse transcriptase, and the cDNA is used for amplification with specific primers. In all eight segments of the IAV gene, the first 12 nucleotides of the 3′ terminus (Uni12) and the first 13 nucleotides of 5' terminus (Uni13) are conservative. Thus, the Uni12 and Uni13 sequences are amplified in a single step by all segments of IAV [54,55]. RT-PCR with Uni12 and Uni13 primers is a powerful tool for detecting IAV in human nasal swabs, and provides accurate information about viral subtypes by means of subsequent sequencing analysis. 

For subtyping of IAV, subtype-specific primers must be designed for amplification by RT-PCR, because of the variable sequences of HA and NA segments. Degenerated primers are suitable for the detection of unknown or newly emerging subtypes of IAV. Rapid identification of this cleavage site figures prominently in the surveillance of avian IAVs. PanHA RT-PCR is a new technique that uses degenerated primers to amplify the cleavage site of the HA_0_ gene of variable subtypes [56]. The degenerated primers contain five sets for amplifying the HA_0_ cleavage sites of all 16 HA subtypes. Direct sequencing of the PanHA RT-PCR products was accomplished for molecular characterization of the HA_0_ cleavage site sequences. Restriction fragment mass fingerprint (RFMF) analysis is a mass spectrometric diagnosis for HA cleavage site [57]. Amplified RT-PCR product containing HA cleavage site is digested with a dedicated restriction enzyme cocktail and the resulting fragments are analyzed by mass spectrometry. Subtyping of the NA genes of IAV has also been performed using degenerate RT-PCR [58,59]. In this method, mixture degenerated primers containing the M13 sequence are used to amplify the fragments of all nine NA subtypes [58]. Although degenerate RT-PCR is required for optimization of degenerated primers, the system utilized by degenerated primers is valuable for the first screening of unknown samples in surveillance.

### 4.2. Quantitative Real-Time PCR

Quantitative real-time PCR (qRT-PCR) is frequently used for detecting and subtyping viruses, including IAVs [60,61]. The method employs molecular oligonucleotide probes, conjugated with a fluorescent molecular or chemical dye for staining the PCR products. One such example is the TaqMan® probe (Applied Biosystems, CA, USA), which is covalently conjugated with a fluorophore and a quencher at the 5′ and 3′ termini, respectively. This results in fluorescent quenching and annealing with the specific region in the PCR products. The PCR primers are extended by Taq polymerase, and the probe annealed with the PCR template is degraded by the 5′ to 3′ exonuclease activity of Taq polymerase. The degradation of the probe causes the separation of fluorophore from the quencher, with the fluorescence detected in a PCR product dose-dependent manner. The SYBR® green method (Applied Biosystems, CA, USA) is a typical qRT-PCR protocol using an intercalator. SYBR green interacts strongly with double-stranded DNA, but only weakly with single-stranded DNA and RNA. The DNA–SYBR green complex expresses green fluorescent signals, and therefore the density of fluorescence is enhanced in proportion to the PCR product dose. Using oligonucleotide probes and intercalators, qRT-PCR offers high sensitivity, specificity, and a wide range of quantification. Thus, it is one of the best techniques for the diagnosis and surveillance of IAVs. The TaqMan approach is commonly used in the diagnosis and surveillance of IAVs (including avian IAVs), because of its high sensitivity and specificity. The matrix gene segment of IAV, which is conservative in different subtypes, is a target of PCR amplification during detecting IAVs [62,63]. The sensitivity of the TaqMan method enables the detection of IAV in infected birds and humans. By contrast, in the SYBR green method, the reaction conditions must be optimized because of the absence of a specific probe. Nevertheless, this lack of necessity for a specific probe offers a simple and inexpensive approach for the diagnosis and screening of IAVs. Moreover, melting curve analysis of the SYBR green method can be used to distinguish subtypes of IAV, and also minor sequence variations of different matrix genes [64,65]. Thus, the SYBR green method is more suitable for high-throughput diagnosis and surveillance than is the TaqMan approach.

Recently, the TaqMan qRT-PCR method was applied specifically to detect *HA* and *NA* genes of the HPAI H5N1 virus. Using the optimized qRT-PCR system, HPAI H5N1 viral RNA was detected and quantified in infected wild birds and humans [66,67]. The sensitivity of the TaqMan method for detecting AI virus is extremely high. Using a specific TaqMan probe conjugated with minor groove binder groups, the minimum detectable level of viral genome was reported to be 0.001 TCID_50_/reaction and 0.08 EID_50_/reaction [68]. Locked nucleic acid (LNA), a high-affinity DNA analog, is suitable for improving the TaqMan probe, because the DNA/LNA copolymers are thermally stable and resistant to exo- and endonucleases [69,70]. qRT-PCR using a LNA-TaqMan probe was developed to detect two different clades (clade 1 and clade 2) of the HPAI H5N1 virus from infected humans [71]. The analytical sensitivity was 10–100 copies/reaction, and the sensitivity was 97% (56 H5N1 HPAI viruses were identified in 58 clinical samples of H5N1 HPAI virus.). Following intranasal inoculation, the HPAI H5N1 virus was detected in chicken meat, including cardiac and skeletal muscle [72]. SYBR-green-based qRT-PCR was also reported to subtype all *HA* and *NA* genes of avian IAVs [73]. Hoffmann *et al.* [74] developed qRT-PCR with specific probes for HA cleavage site. The probe FliH5-CS-FAM was designed to identify HA cleavage site of HPAI H5N1 virus Qinghai lineage and the qRT-PCR detected HPAI Qinghai-like viruses or very closely related isolates of Asian origin. These results indicate the importance of qRT-PCR in the clinical investigation and surveillance of the HPAI H5N1 virus. 

**Figure 2:**
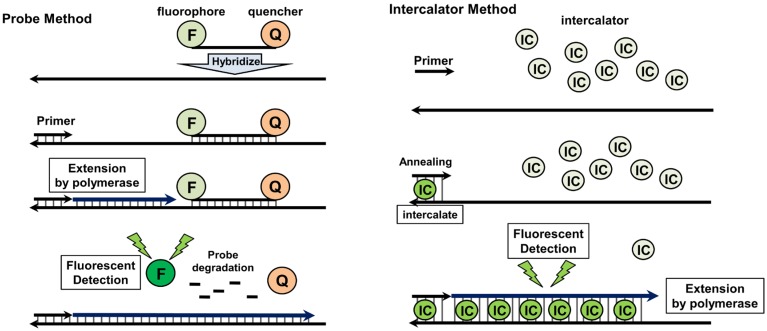
Schematic diagram of qRT-PCR.

**Figure 3:**
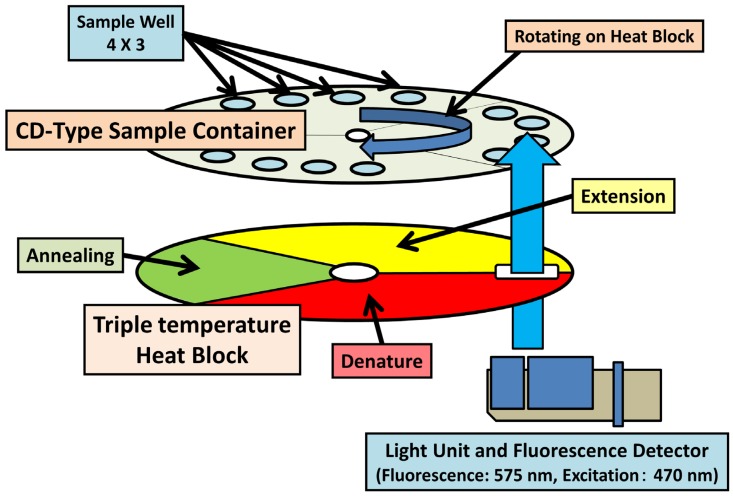
Schematic diagram of SHRT-PCR.

qRT-PCR offers high sensitivity and selectivity, but generally requires approximately 2 h per run, which is longer than the time required for rapid diagnosis systems such as IC. Super high-speed qRT-PCR (SHRT-PCR) is a recently developed version of qRT-PCR, characterized by an extremely short reaction time (less than 20 min per run for 40 cycles) [75]. The reaction mixtures of qRT-PCR are applied to thin compact disc (CD)-type sample containers, sealed, and rotated on heat blocks at three different temperatures (Figure 3). The unique structural and thermodynamic properties of heat blocks fixed at three different temperatures are critical for the super high-speed polymerase chain reaction, because the blocks allow rapid temperature changes within the samples. The SHRT-PCR system detects viral RNA segments of IAV, including the H5N1 subtype, as efficiently as conventional RT-PCR methods, and therefore offers considerable advantages in the rapid diagnosis and detection of infections. Despite these advantages, the SHRT-PCR system is limited to a sample capacity of 12. Given that a quantitative assay requires multiple defined amounts of sample to generate a standard curve, SHRT-PCR may be more suited to performing qualitative rather than quantitative assays. Thus, the system represents a useful alternative to IC, for rapid diagnosis in local clinics aimed at containment of infected patients. Nevertheless, the improved reaction speed of SHRT-PCR is expected to lay the foundation for a “new generation” qRT-PCR approach in the future.

Multiplex PCR is another variant of RT-PCR or qRT-PCR, characterized by multiple DNA amplification, with multiple sets of primers, in a single tube. The system has the advantage of high-throughput typing and subtyping during the surveillance of IAVs. Multiplex RT-PCR [76,77] and multiplex qRT-PCR [78,79,80,81] have been extensively used for detecting avian IAVs, including the HPAI H5N1 virus. Suwannakarn *et al.* [82] developed multiplex qRT-PCR with TaqMan probes for typing and subtyping IAVs. Wang *et al.* [83] used multiplex qRT-PCR with SYBR green for subtyping of H5 subtypes, based on cost-effectiveness. FluPlex is a multiplex RT-PCR enzyme hybridization assay [84], reported to be capable of typing influenza viruses and subtyping HA (H1, H2, H3, H5, H7, and H9) and NA (human N1, animal N1, N2, and N7) proteins. Thus, almost all subtypes of IAVs isolated from humans are covered by multiplex PCR. The system exhibits high sensitivity (10–100 copies/reaction), and therefore plays an important role in typing and subtyping aimed at high-throughput and high-sensitivity surveillance.

## 5. DNA Amplification under Isothermal Conditions

### 5.1. Loop-Mediated Isothermal Amplification

**Figure 4:**
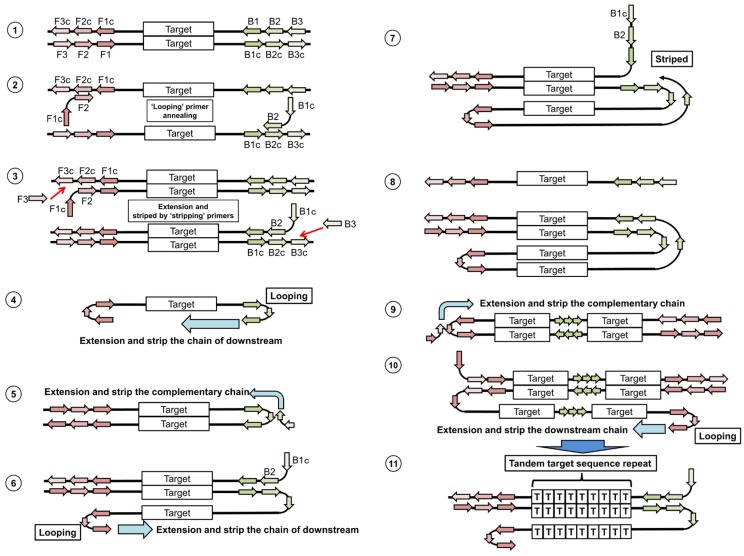
Schematic diagram of Loop-mediated isothermal amplification (LAMP).

Loop-mediated isothermal amplification (LAMP) is similar to the PCR-based method, but uses DNA amplification under isothermal conditions [85]. The system employs a DNA polymerase and four primers, including two “looping primers” and two “stripping primers”. First, the looping primers anneal on the F2 or B2 regions, and complementary DNA chains are amplified (Figure 4, step 2). Next, the stripping primers anneal on the F3 or B3 regions, and complementary DNA chains are amplified. This leads to the release of the complementary DNA chains generated by the looping primers (Figure 4, step 3). Finally, the single chain containing both terminals makes stem-loops (Figure 4, step 4). In the 5′ terminal, the double-stranded F1 or B1 region works as a primer, resulting in the generation of a double-stranded chain containing a stem-loop (Figure 4, step 5). The stem-loop area (F2 or B2 region) is a single-stranded chain, and therefore new looping primers can anneal on the F2 or B2 region. A new complementary chain is generated from the looping primer (Figure 4, step 6), and strips the corresponding chain (Figure 4, step 7 and 8). Similar to step 4, the 5′ terminal makes a stem-loop, and the new chains are generated (Figure 4, step 9). The extensions from the F2 or B2 region, and the extension formed by the looping primer on the stem-loop area, occur alternately (Figure 4, steps 9 and 10). Finally, the long reaction products containing tandem target sequence repeats are generated (Figure 4, step 11). 

Reverse transcription LAMP (RT-LAMP) is an improved LAMP suitable for RNA detection. Reverse transcriptase (RTase) is added for reverse transcription performed by using looping primers or stripping primers. RT-LAMP has been used to detect the HPAI H5N1 virus [86,87], and other IAV subtypes [88,89,90]. RT-LAMP was comparable to that of the RT-PCR method currently recommended by WHO [87]. For detection of the avian IAV H9 subtype, the sensitivity of RT-LAMP was 10-fold higher than that of RT-PCR [90]. In the other hand, two commercial RT-LAMPs showed a lower sensitivity to detect several HPAI H5 and H7 viruses, including Southeast Asian HPAIV strains, than qRT-PCR [91]. This report indicates difficulty in optimizing LAMP primers with broad reactivity and specificity. RT-LAMP has also been used to identify IAV H1 and H3 subtypes, and to detect influenza B virus [86]. Nagatani *et al.* [92] monitored the amplified DNA by RT-LAMP, using a small and convenient portable potentiostat, with methylene blue as an intercalator. Although optimization of primer design is required for high sensitivity and specificity, the simplicity of LAMP makes it suitable for field surveillance and diagnosis in developing countries.

### 5.2. Nucleic Acid Sequencing-Based Amplification/Real-Time Nucleic Acid Sequencing-Based Amplification

**Figure 5:**
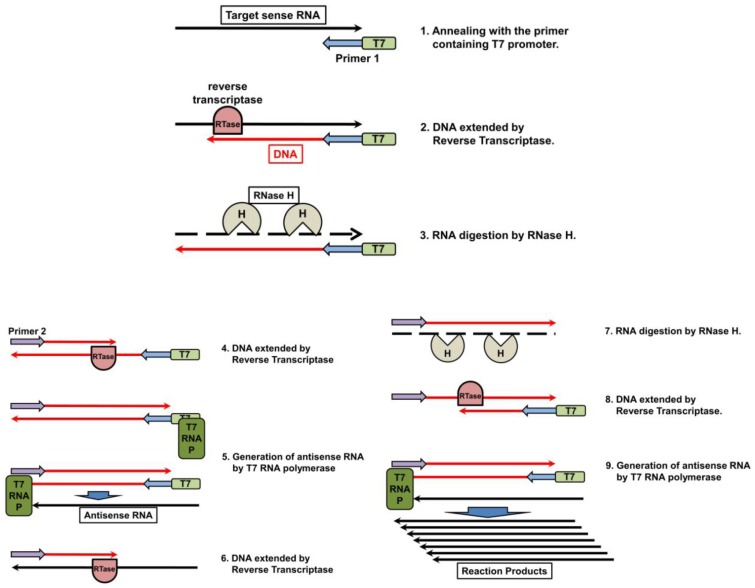
Schematic diagram of nucleic acid sequencing-based amplification (NASBA).

Nucleic acid sequencing-based amplification (NASBA) is an isothermal nucleic acid amplification system, based on transcription and reverse transcription [93]. The method utilizes RTase, RNA polymerase, RNaseH, a primer containing T7 promoter (primer 1), and a reverse primer (primer 2). First, primer 1 anneals with target sense RNA, and RTase extends the complementary DNA (Figure 5, steps 1 and 2). RNaseH recognizes the DNA–RNA hybrids, and only digests the RNA chain (Figure 5, step 3). RTase uses its DNA polymerase activity to generate double-stranded DNA with primer 2 (Figure 5, step 4). The interaction of T7 RNA polymerase with T7 promoter on the double-stranded DNA generates antisense RNA (Figure 5, step 5). Using this antisense RNA as a template, RTase generates a DNA chain (Figure 5, step 6). RNaseH digests the RNA (Figure 5, step 7), and RTase generates double-stranded DNA (Figure 5, step 8). Finally, considerable quantities of antisense RNA are generated as reaction products (Figure 5, step 9). 

NASBA is suitable for the detection of RNA viruses such as IAVs, because RNAs are utilized as amplification templates. The technique has been used to detect the *HA* gene of AI virus subtypes H5 [94,95] and H7 [96], and also the matrix gene [97], which is the consensus of all IAV subtypes. Real-time NASBA is a variant of NASBA, characterized by the use of a fluorescent-labeled probe or intercalator, and allowing a quantitative measurement. The limit of analytical detection of real-time NASBA for detecting an HPAI H5N1 virus isolated from animals was reported to be ≥10 copies/reaction. By contrast, while TaqMan assay appeared to be less sensitive at approximately 100 copies/reaction) [98]. In clinical samples obtained from seven confirmed human cases of HPAI H5N1 virus infection, the level of sensitivity of NASBA was 10 TCID_50_/mL [99]. Recently, real-time NASBA was used for the detection of IAV H1N1 and H3N2 subtypes, influenza B virus, respiratory syncytial virus (RSV), and metapneumovirus isolated from the swabs of patients with respiratory tract infections [100]. Multiplex NASBA is an improved method, which is suitable for the simultaneous quantification of multiple target genes [101]. Multiplex NASBA with an enzyme-linked oligonucleotide capture optical detection method can distinguish between a group of viruses causing common lower respiratory tract infections, including influenza A and B viruses, human parainfluenza virus 1-4, RSV, rubella virus, and Coxsackie virus [102]. The high sensitivity and specificity of NASBA makes it one of the most powerful tools for detection, surveillance typing and subtyping, laboratory research, and clinical investigation. Furthermore, similar to LAMP, the technique does not require expensive instruments such as thermal cyclers and fluorescent detectors. As such, isothermal detection systems are expected to play an important role in field surveillance and diagnosis in developing countries.

## 6. DNA Microarray

DNA microarray is a collection of oligonucleotide, probes, or DNA spots immobilized on a solid surface. The technology is applied to high-throughput and simultaneous wide genomic screening [103,104]. DNA microarray also plays a critical role in medical diagnosis and surveillance of infectious diseases, including IAV [105,106,107]. FluChip-55 is a microarray used for typing and subtyping IAVs. FluChip-55 recognizes 55 influenza viral sequences, including the HA genes of subtypes H1, H3, and H5, and type B; the NA genes of subtypes N1 and N2; the M genes of types A and B; and the NP gene of type B [108,109]. Huang et *al* [110] developed a microarray simultaneously to type influenza A and B viruses, and to distinguish between subtypes H1N1 and H3N2 of the AI H5N1 virus. Recently, a universal oligonucleotide microarray for near-complete subtyping of IAVs was reported [107]. The microarray contains two sets of oligonucleotide probes, for classification of the subtypes of hemagglutinin (H1–H13, H15, and H16) and neuraminidase (N1–N9). By contrast, MChip is an unique microarray for typing and subtyping, characterized by identification of only the M gene segment of IAV [111,112]. MChip utilizes only 15 short oligonucleotides for hybridization. Several target sequences were chosen to distinguish between the different subtypes of IAV, while others were selected to identify a wide range of IAV subtypes. The pattern of fluorescence signal intensity of each array provides information on the IAV subtype. MChip was successfully employed in the identification of >100 AI H5N1 viruses and 16 historic H1N1 IAVs, including regenerated Spanish influenza virus A/Brevig Mission/1/1918 [112,113]. The use of DNA microarray is restricted, because of the requirement for expensive data collection instruments and specialized data analysis techniques. In addition, a microarray requires a certain amount of cDNA, which implies that the virus sample may have to be propagated in embryonated chicken eggs or culture cells. Nevertheless, the technique is one of the most powerful methods for detecting IAVs with extremely high-throughput.

**Figure 6:**
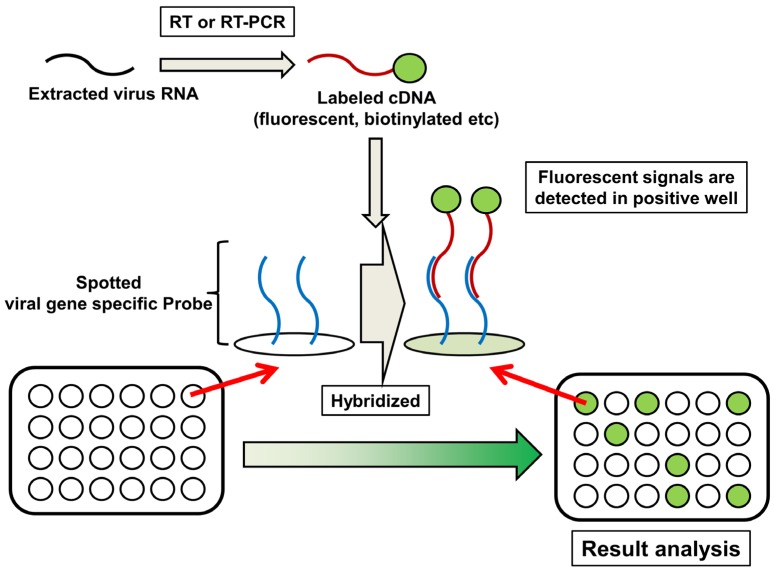
Schematic diagram of microarray.

## 7. Concluding Remarks

The frequent outbreaks of HPAI H5N1 viruses in the last decade have dramatically accelerated the development of diagnosis and surveillance methods. In 2009, this process was highlighted by the spread of the first influenza pandemic of the 21^st^ century, caused by swine-origin influenza virus A/H1N1pdm, throughout Mexico and the southwestern USA. Global society is increasingly aware of the need for molecular diagnosis and surveillance of IAVs, in advance of newly emerging pandemics. A wide variety of methods for detecting HPAI H5N1 viruses have been developed worldwide (Table 1). 

IC and improved IC (using silver amplification or fluorescent beads) can detect viral antigen within 30 minutes without specific preparation. However, the easy and rapid diagnosis is less sensitive than other methods presented in the review (See chapter2). In addition, IC is based on antigen-based principle like ELISA, meaning IC could be low sensitivity for antigenic related emergent H5 or H7 IAVs. Thus, IC is suitable for rapid check in small amount of samples or diagnosis in clinics and small hospitals. Although PCR-based methods are expected to be new gold standards, there are some advantages and disadvantages in each method. RNA extraction, which is usually a time consuming step, is one of disadvantages in these methods. However, the new technologies of RNA extraction systems provide prospects for saving time in the PCR-based diagnoses. (See chapter 3). Specificity versus sensitivity is a difficult problem, because high specific detection system would miss to detect new emerging virus. The primers and probes are designed in conservative regions yet mismatches with emerging sequences could occur. Although degenerate and multiplex PCR can detect a wide range of viral genome mutation, these primers easily cause non-specific amplification. Thus, the combination of initial screen using degenerate and multiplex PCR and specific diagnosis using qRT-PCR is preferable to detection of HPAI H5N1 and H7N7 viruses. PCR-based methods require approximately 2 hours for PCR. SHRT-PCR is characterized by an extremely short reaction time (less than 20 minutes). The combination of SHRT-PCR and TruTip (See chapter 3) is comparable to IC in amount of time required, however SHRT-PCR remains at laboratory stage because of the limited capacity of samples and immature usability. Although PCR-based methods can identify the subtypes of H5 and H7 viruses, a portion of H5N1 viruses and a certain level of H7N7 viruses are not HPAI but LPAI viruses. For detection of HPAI viruses, cleavage site of HA should be analyzed. Sequencing or mass spectrometric assay for amplified PCR product containing HA cleavage site is suitable for characterization of HA cleavage site (see chapter 4.1). However, these methods require the additional expensive installments. Although qRT-PCR with specific probes for HA cleavage site is capable of differentiation between HPAI and LPAI viruses, the high specificity could miss other linages of HPAI viruses (see chapter 4.2). RT-LAMP and NASBA show great potential for IAVs detection in developing countries and field surveillance, because isothermal instruments can be cheap and mobile. If these methods are used under such rough conditions, requirement of RNA extraction is a bigger problem than the case of PCR-based method. Microarray is potentially a great powerful tool for detecting IAVs with extremely high-throughput, however the equipment is expensive and the analysis is complex. Thus, the system is suitable for high-level laboratory or national core facility. Although some of these techniques are expected to become gold standards for the diagnosis of IVAs, many remain at the laboratory stage. Further optimizations and validations are required before such methods are suitable for clinical and surveillance applications. Moreover, continuous upgrades in the diagnosis of IAV are required, because of frequent reassortment. Nevertheless, the methods we reviewed can offer the best prospects for providing critical information useful for prevention and control of newly emerging pandemic viruses.

**Table 1:** Comparison of diagnosis methods for influenza A virus

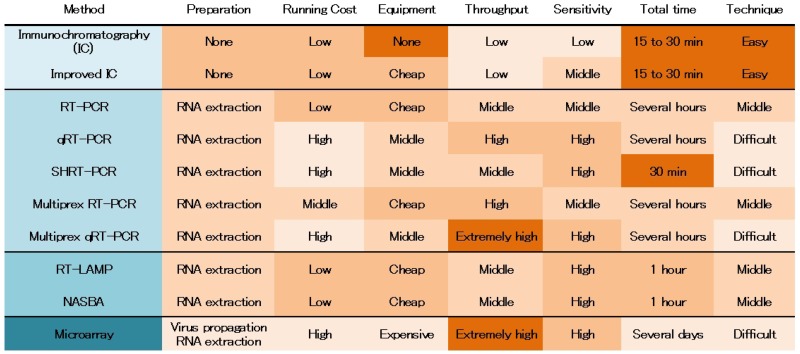

Improved IC:IC with silver amplification or fluorescent beads, RT-PCR: reverse transcription PCR, qRT-PCR: quantitative real-time PCR, SHRT-PCR: super high speed real-time PCR, RT-LAMP: reverse transcription Loop-mediated isothermal amplification, NASBA: Nucleic acid sequencing-based amplification Acceptable specimens (not limited to): virus isolates; nasal, throat and cloacal swab; nasal bronchial and throat wash.
